# Design and Characterization of Bioengineered Cancer-Like Stem Cells

**DOI:** 10.1371/journal.pone.0141172

**Published:** 2015-10-21

**Authors:** Sungpil Cho, Hongsuk Park, Elke A. Jarboe, C. Matthew Peterson, You Han Bae, Margit M. Janát-Amsbury

**Affiliations:** 1 Department of Obstetrics and Gynecology, Division of Gynecologic Oncology, Salt Lake City, Utah, United States of America; 2 Department of Bioengineering, Salt Lake City, Utah, United States of America; 3 Department of Pathology, Salt Lake City, Utah, United States of America; 4 Department of Pharmaceutics and Pharmaceutical Chemistry, University of Utah, Salt Lake City, Utah, United States of America; Okayama University, JAPAN

## Abstract

Cancer stem cells (CSCs) are a small subset of cancer cells responsible for maintenance and progression of several types of cancer. Isolation, propagation, and the differentiation of CSCs in the proper stem niches expose the intrinsic difficulties for further studies. Here we show that induced cancer like stem cells (iCLSCs) can be generated by *in vitro* oncogenic manipulation of mouse embryonic stem cells (mESCs) with well-defined oncogenic elements; SV40 LTg and H*ras*V12 by using a mouse stem virus long terminal repeat (MSCV-LTR)-based retroviral system. The reprogrammed mESCs using both oncogenes were characterized through their oncogenic gene expression, the enhancement of proliferation, and unhampered maintenance of stem properties *in vitro* and *in vivo*. In addition, these transformed cells resulted in the formation of malignant, immature ovarian teratomas *in viv*o. To successfully further expand these properties to other organs and species, more research needs to be done to fully understand the role of a tumor- favorable microenvironment. Our current study has provided a novel approach to generate induced cancer like stem cells through *in vitro* oncogenic reprogramming and successfully initiated organ-specific malignant tumor formation in an orthotopic small animal cancer model.

## Introduction

The hierarchical theory of the organization of cancer suggests that only a small subset of cells is responsible for the initiation and further growth of cancer [1–3]. Those small populations of cells have been defined as cancer stem cells (CSCs). Amongst others, CSCs exhibit features such as self-renewal and the ability of differentiation into heterogeneous and tumorigenic cancer cells [1, 4]. Putative CSCs from various tumors including brain, breast, and ovarian cancer were isolated to date based on their expression of specific molecules or combination of cellular markers (e.g. CD133, CD44, ALDH) [5–9]. The tumorigenic potential of these cells has been demonstrated in various xenograft studies using immune compromised mice [5, 6, 10]. However, further characterization of CSCs’ properties and capabilities have been hampered by intrinsic difficulties of isolating pure CSCs populations, propagation of these isolated CSCs, and the differentiation of CSCs in the proper stem niches [11].

Normal fibroblast and breast cells can be transformed into their induced cancer cells by *in vitro* reprogramming through the exogenous introduction of genetic alternations responsible for increasing the length of telomere (hTERT), providing constitutive proliferation signals (H*ras*V12), and inhibiting growth suppressor pathways such as p53 and pRB by simian virus 40 (SV40) antigens [12, 13]. Along the same lines of *in vitro* reprogramming, Scaffidi *et al* reported that somatic cells possess enough plasticity to be reprogrammed and acquire CSC properties through *in vitro* oncogenic introduction [14]. Numerous references, especially in the study of hematological cancers, indicated that CSCs could be derived from tissue stem, progenitor cells, and even from somatic cells [10, 14–18]. However, the potential of *in vitro* reprograming of embryonic stem cells into CSCs has remained unclear.

Herein, we studied whether mouse embryonic stem cells (mESCs) can be successfully reprogrammed into induced cancer like stem cells (iCLSCs) through *in vitro* oncogenic manipulation. In addition, by exposing iCLSCs to various specific microenvironments *in vivo*, we could observe the potential of these iCLSCs to generate site-specific iCLSC tumors in immune competent mice.

## Materials and Methods

### Chemicals and Plasmids

The following materials were obtained from the manufacturers indicated: (a) Fugene 6 (Promega, Madison, WI), (b) Polybrene and FBS (Sigma-Aldrich, St-Louis, MO), (c) Geltrex (Invitrogen, Carlsbad, CA) (d) Mycozap plus CL (Lonza, Allendale, NJ), (e) pBABE-H*ras*V12 (#1768), pBabe-SV40 LTg (#10891), pMSCV-GFP (#33336), and pMSCV-RFP (#33337) (Addgene, Cambridge, MA), (f) pVSV-G (Clontech, Mountain view, CA).

### Cell Culture

GP2-293 cells (Clontech) and their derivatives, and γ-irradiated mouse embryonic fibroblast (mEF) cells (Cyagen, Santa Clara, CA) were maintained in DMEM (ATCC, Manassas, VA) supplemented with either 10% or 15% fetal bovine serum (Sigma-Aldrich,), respectively. Mouse embryonic stem cells (mESCs) (C57BL/6 mouse embryonic stem cells, # MUBES-01001, Cyagen) were maintained in Knockout DMEM (Invitrogen) supplemented with 15% knockout serum replacement (Invitrogen), 1% L-glutamine (Invitrogen), 1% Non-essential amino acids (Invitrogen), 0.1% beta-mercaptanol (Invitrogen), Leukemia inhibitory factor (LIF, 10 ng/ml in culture media; StemRD, Burlingame, CA). All cells were maintained in a humidified incubator with 5% CO_2_ atmosphere at 37°C.

### Sub-Cloning of Genes to pMSCV

To perform sub-cloning, H*ras*V12 and SV40 large T antigene (LTg) were separated from pBABE-H*ras*V12 and pBABE-SV40 LTg by the enzymatic digestion with BamHI and EcoRI (NEB, Ipswich, MA), or with BamHI (NEB), respectively. Integration of H*ras*V12 and SV40LTg into either pMSCV-GFP or pMSCV-RFP was performed by ligation with T4-ligase (NEB) and produced pMSCV-H*ras*V12-GFP and pMSCV-SV40 LTg-RFP. Integration of the inserts was verified by both enzymatic digestion and DNA sequencing.

### Generation of Retrovirus

To generate retroviral supernatant, GP2-293 cells were transiently transfected with either pMSCV-H*ras*V12-GFP or pMSCV-SV40 LTg-RFP combined with the replication-incompetent helper vector pVSV-G in a 60 mm culture dish by using Fugene 6. The cells were fed at 24 h post-transfection, and retroviral supernatant was pooled from the collection at 48 and 72 h post-transfection. The supernatant was aliquoted after centrifugation and stored at –80°C for future use. The viral titer was verified by FACS analysis (FACSCanto Analyzer, BD Biosciences, San Jose, CA) after infection to 1x 10^5^ NIH-3T3 (ATCC) mouse fibroblast cells.

### Establishment of Stable GP2-293 derivatives

To generate GP2-293 derivatives with stable gene integration, GP2-293 cells were either infected with H*ras*V12 or SV40 LTg using a retroviral system. FACS sorting was performed to select stably infected cells according to GFP or RFP expression (FACSAria cell sorter, BD Biosciences, San Jose, CA). The gene expression in stable cells was verified by western blot.

### Retroviral Infection to mESCs

mESCs were washed and trypsinized. After centrifugation, they were re-suspended in serum free mESCs medium containing 8 μg of Polybrene per ml—PBS and plated at 1x10^5^ cells / 1ml per well of a twelve-well plate. Retroviral supernatant from single virus was added at 1 ml / 1x10^5^ cells or same volume ratio of retroviral supernatant from individual virus was added for co-infection. After 1hr incubation in the CO_2_ incubator, the plate was centrifuged for 2 h at 1,000 g at room temperature. Next day, the retroviral supernatants were removed; the cells were re-suspended in mESCs medium and plated onto dishes either coated with Geltrex or plated with irradiated mEF. The stably infected mESCs were sorted by FACS based on GFP or RFP expression (FACSAria cell sorter).

### Western Blotting

The cells were lysed with RIPA buffer (#9806, Cell Signaling) supplemented with 1mM PMSF (Sigma-Aldrich). Western blotting was performed using 4–20% gradient SDS-polyacrylamide TRIS-HCl gel (Mini-PROTEAN TGX^®^, BioRad) according to the manufacturer’s protocol (Biorad). Antibodies used for western blotting were anti-ras (#610001, 1:1000, BD Bioscience), anti-SV40 large T and small t antigen (#554150, 1:1000, BD Bioscience), anti-β-actin (#A5441, 1:2500, Sigma-Aldrich), and anti-mouse IgG-HRP (#7076S, 1:5000, Cell Signaling).

### Alkaline Phosphatase Staining and Immunocytochemistry Staining of Live Cells

Alkaline phosphatase staining was performed using the alkaline phosphatase staining kit II according to the manufacturer’s protocol (Stemgent). For immunocytochemistry staining of live cells, cells cultured in a 12 well plate until showing visible colonies were treated with antibody (StainAlive™ SSEA-1 (DyLight 550™), Stemgent) solution prepared in fresh cell culture medium with a final concentration of 2.5 μg/ml. After incubation for 30 min at 37°C and 5% CO_2_, cells were examined under a fluorescent microscope (automated microscope, Nikon, Japan) with TRITC filter.

### Cell Proliferation Assay

Cell proliferation assay was performed by utilizing cell counting assay kit-8 (CCK-8, Dojindo, Rockville, MD). Briefly, the 100 μl of cell suspension (3000 cells / well) was plated in the 96 well culture plate coated with Geltrex (Invitrogen). During the growth period, cell proliferation in each well was measured after 1 h incubation with 10 μl of CCK-8 solution using a microplate reader (Spectramax 250, Molecular Device Inc., Sunnyvale, CA) at a wavelength of 540 nm. After subtraction of intrinsic absorbance of media at a wavelength of 540 nm, the relative cell proliferation (%) was calculated from ([Absorbance] _test_ / [Absorbance] _control_) × 100. [Absorbance] _control_ refers to the absorbance of cells cultured in media at day 1 after seeding of cells to the plate.

### In vivo tumor production utilizing modified mESCs and histo-pathological tissue evaluation

This study was carried out in strict accordance with the recommendations in the Guide for the Care and Use of Laboratory Animals of the National Institutes of Health. The protocol was approved by the Committee on the Ethics of Animal Experiments of the University of Utah (Permit Number: 14–08011).

Both, modified mESCs and naive (control) mESCs were cultured for one week on mEF feeder cells until visible colonies were seen prior implantation. For orthotopic ovarian bursa inoculation of cells to animal, six to eight week old, female C57BL/6 mice (Jackson lab, Bar harbor, ME) were weighed before intraperitoneal anesthetic injections (xylazein-ketamine; 0.1 ml/10 g body weight). Each animal placed in prone position was received about ~1.5 cm in length of dorsal incision, slightly to the left of midline. The dermis was separated from underlying tissues, and a smaller incision was made through the dorsal fascia flat muscle to access the abdominal peritoneum. After identifying left kidney, the area immediately below the kidney was dissected to locate the left ovary, which was then externalized through the incision. 1x 10^5^ cells (50 μl in 1:1 mixture of PBS and matrigel (# 354248, Corning, Tewksbury, MA)) were directly injected into ovarian bursa using a Hamilton syringe (30 G needle). After cell inoculation, the ovary was place back to its original position into the peritoneal cavity. 4–0 sutures were used to suture incision of the back wall and skin [19, 20]. Orthotopic inoculation of cells to breast of mice was performed by direct injection of 1x 10^5^ cells (50 μl in 1:1 mixture of PBS and matrigel (# 354248, Corning, Tewksbury, MA)) to the right bottom mammalian fat pad.

This pilot *in vivo* study included (n = 16; S1 Table) animals. In brief, depending on the tumor site (mammary gland versus ovarian bursa), either immature teratomas with malignant properties (ovary) or mature teratomas (breast) formed. The total number of animals (n = 16) were divided into four experimental groups. Group1: Mammary gland inoculated with mESC; Group 2: Ovarian bursa inoculated with mESC; Group 3: Mammary gland inoculated with mESC-Ras-LTg (iCLSCs), Group 4: Ovarian bursa inoculated with mESC-Ras-LTg (iCLSCs). After orthotopic cell inoculation, mice were monitored bi-weekly throughout the entire 15 week experimental periods. Animals were housed under standard conditions in the Center for Comparative Medicine Animal Facility in accordance with guidelines of the Institutional Animal Care and Use Committee (IACUC) at the University of Utah. For the histo-pathological evaluation of tissues, hematoxylin and eosin (H&E) stains were performed on representative sections of tumor mass by ARUP laboratory (ARUP, Salt Lake City, UT). A pathologist with gynecologic oncology specialization evaluated digital microscopic images.

### Statistical analysis

Statistical analysis and plotting of graphs were performed using GraphPad Prism software (GraphPad Software Inc., San Diego, CA, USA). All of the results are expressed as the mean ± SD, and p<0.05 was used for statistical significance.

## Results

### Construction of pMSCV-HrasV12 and pMSCV-LTg

**Fig 1:**
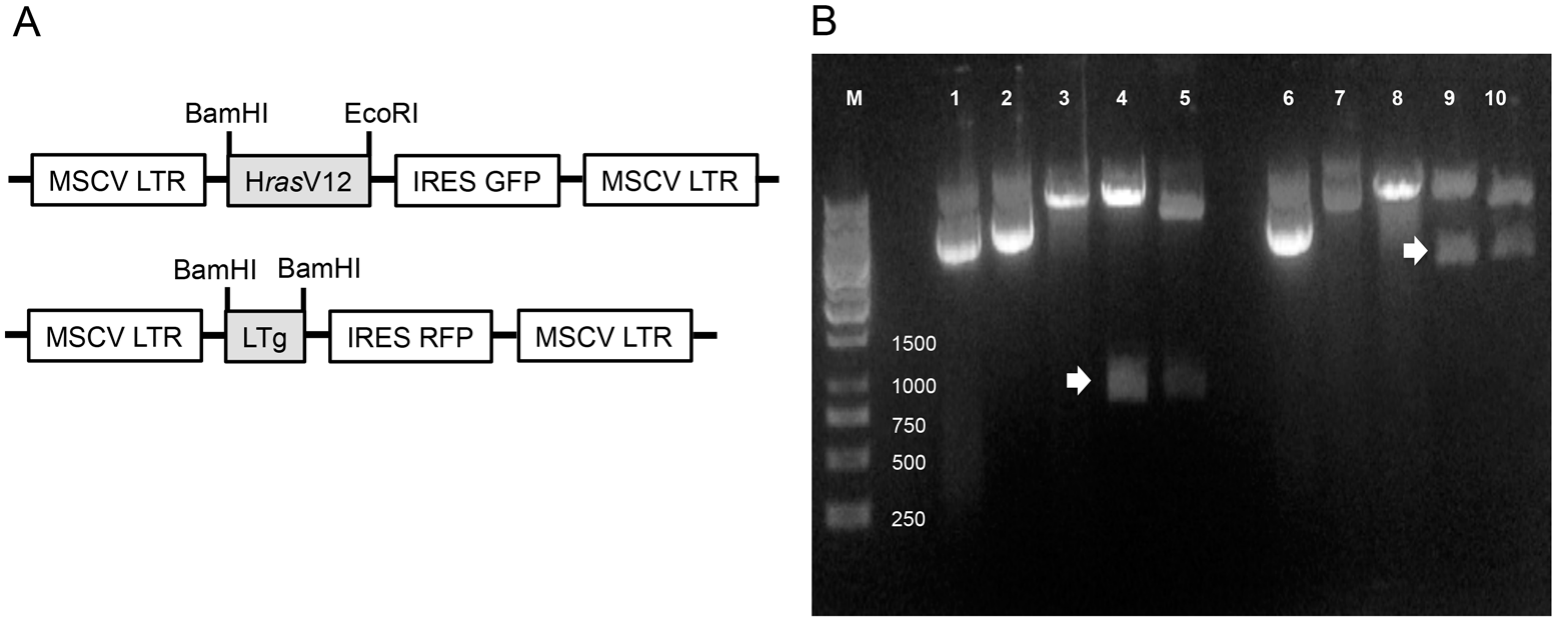
Sub-cloning of H*ras*V12 and LTg into pMSCV plasmids. (A) Genes of interest (i.e. HrasV12 or LTg) were inserted in between MSCV LTRs, and either GFP or RFP gene was used as a tracer gene. (B) Inserts cloned into pMSCV plasmids were confirmed by enzymatic digestions with either BamHI or EcoRI. M: DNA ladder, 1: pMSCV-GFP; 2: pMSCV-H*ras*V12-GFP; 3: pMSCV-GFP^cut^; 4: pMSCV-H*ras*V12-GFP^cut^; 5:pBABE-H*ras*V12^cut^ (+ control); 6: pMSCV-RFP; 7: pMSCV-SV40 LTg-RFP; 8: pMSCV-RFP^cut^ 9: pMSCV-SV40 LTg-RFP^cut^; 10: pBABE-SV40 LTg^cut^ (+ control). White arrows indicate inserts. Sequences of insert were also verified by DNA sequencing.

Retroviral plasmids with the MSCV LTR (mouse stem cell virus long terminal repeat) were constructed through sub-cloning of either H*ras*V12 or SV40 LTg gene into a multiple cloning site (MSC) followed by an IRES driving expression of the GFP or RFP gene shown as schematic diagram in Fig 1A. The inserts in constructs were verified through enzymatic digestion (Fig 1B), and DNA sequencing.

### Establishment of GP2-293 derivatives to generate retrovirus

**Fig 2:**
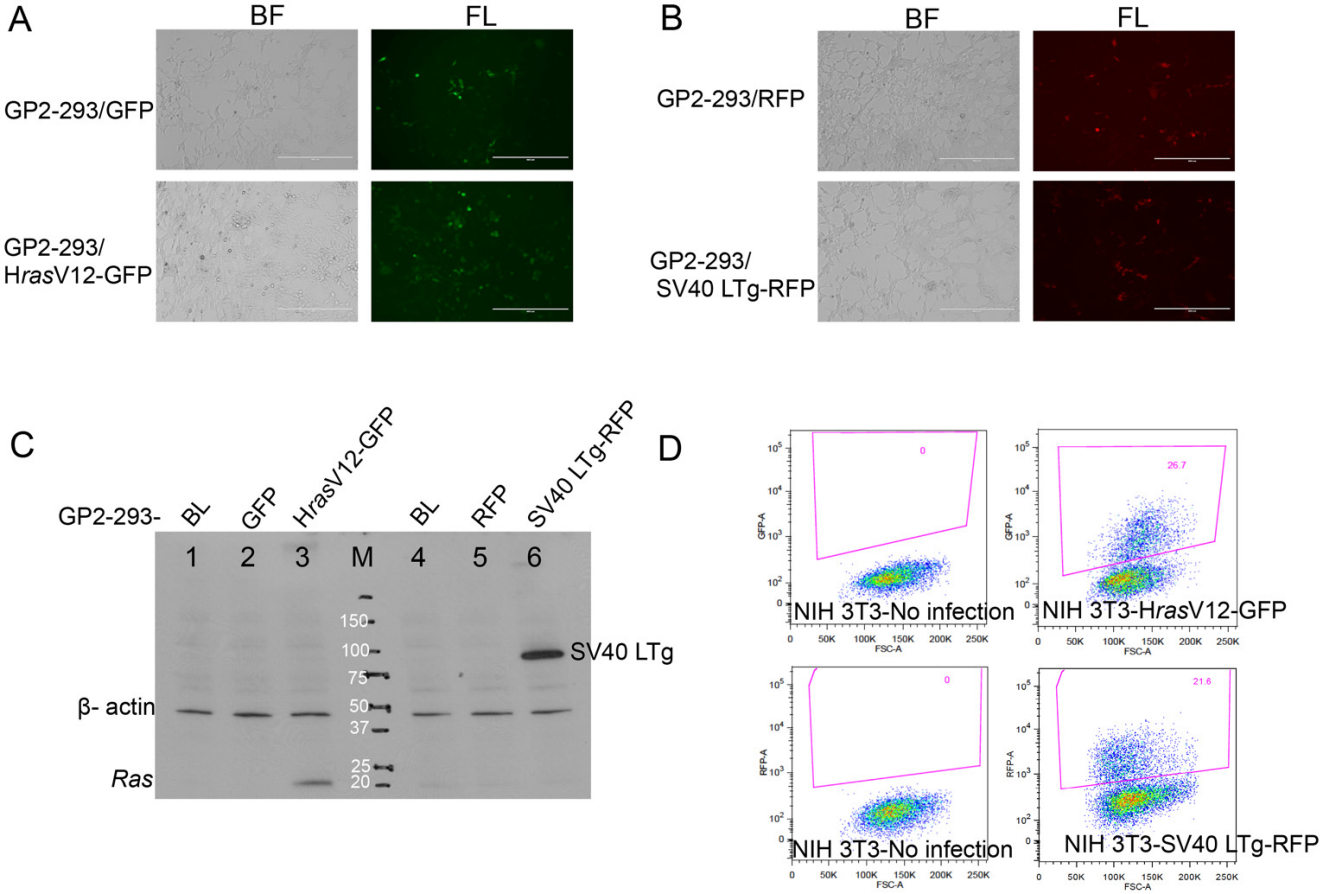
Establishment and characterization of GP2-293 derivatives for stable generation of retrovirus. (A) Confirmation of successful GFP expression in GP2-293 cells, and (B) Confirmation of successful RFP expression in GP2-293 cells after introduction of pMSCV plasmids. BF: bright field image; FL: fluorescence image. Scale bare is 400 μm. (C) Immunoblot analysis illustrating stable expression of H*ras*V12 and SV40 LTg in GP2-293 cell derivatives; 1. BL: GP2-293 blank cell; 2. GFP: GFP containing GP2-293 cell 3. HrasV12-GFP: H*ras containing* GP2-293 cell; 4. BL: GP2-293 blank cell; 5. RFP: RFP containing GP2-293 cell 6. SV40LTg-RFP: SV40LTg and RFP containing GP2-293 cell; M: Protein ladder. (D) Flow cytometry analysis (FACS) of 1x 10^5^ NIH-3T3 mouse fibroblast after infection with retrovirus produced from GP2-293 derivatives.

To establish stable cell lines producing retrovirus, GP2-293 cells, a derivative of 293 human kidney cell line with stable integration of *gag/pol* retroviral components, were transduced with pMSCV plasmids. The cells expressing either GFP or RFP sorted by FACS (Fig 2A and 2B) were further verified to observe their corresponding gene expressions with immunoblot analysis shown in Fig 2C. The verified stable cells were further used to generate retroviruses through transfection of a viral envelope plasmid, pVSV-G. Infection ability of those retroviruses was demonstrated by FACS analysis through the measurement of the amount of retrovirally infected-NIH3T3 mouse fibroblast cells expressing either GFP or RFP (Fig 2D).

### Generation of genetically modified mESCs

**Fig 3:**
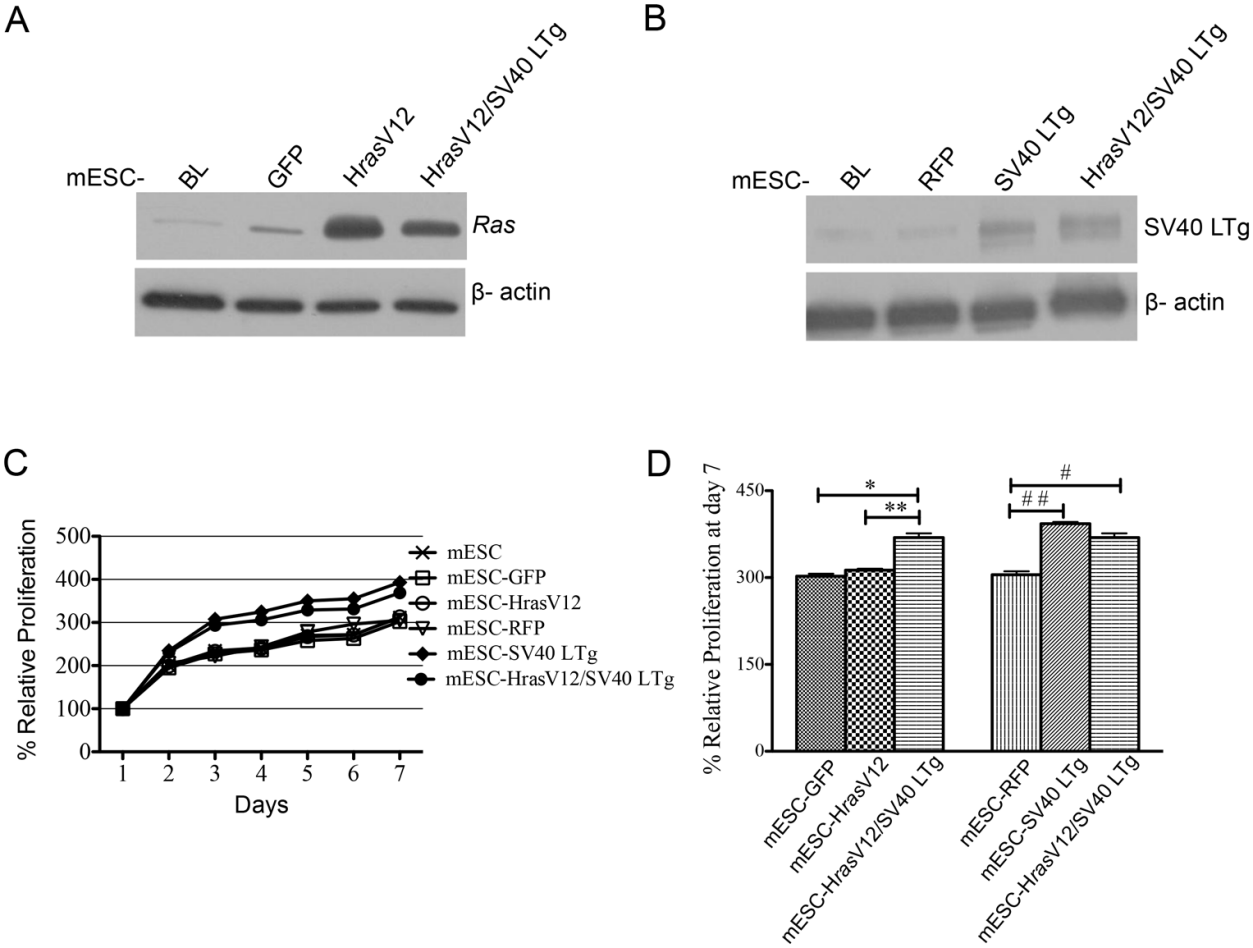
Characterization of genetically modified, retrovirally transduced mESCs. Representative images from immunoblot analysis: (A) H*ras*V12, (B) SV40 LTg. (C) CCK cell proliferation assay for 7 days of proliferation period of mESCs and transformed mESCs. (D) Comparison of proliferations at day 7. Mean ± S.D. (n = 3), *, **, and #, ## p<0.05. ANOVA test was performed with Tukey’s post-test using the GraphPad Prism software.

Mouse embryonic stem cells (mESC) were transformed by infection with retroviruses produced from stable GP2-293 cell derivatives. Expression of genes introduced into mESCs was verified by immunoblot analysis (Fig 3A and 3B). To confirm the changes of cell proliferation, cell proliferation assays were performed on transformed mESCs, which were also compared to control mESCs (Fig 3C). mESC-H*ras*V12/SV40 LTg showed significant increase in proliferation when compared to mESC-GFP and -H*Ras*V12 at Day 7 (Fig 3D). Further comparing proliferation of mESC-SV40 LTg and mESC-H*ras*V12/SV40 LTg with mESC-RFP, mESC-SV40 LTg and mESC-H*ras*V12/SV40 LTg showed significant enhancement of proliferation (Fig 3D). However, there was no difference in proliferation between mESC-H*ras*V12/SV40 LTg and mESC-SV40 LTg (Fig 3D).

### Maintenance of stemness after retroviral modification of mESCs

**Fig 4:**
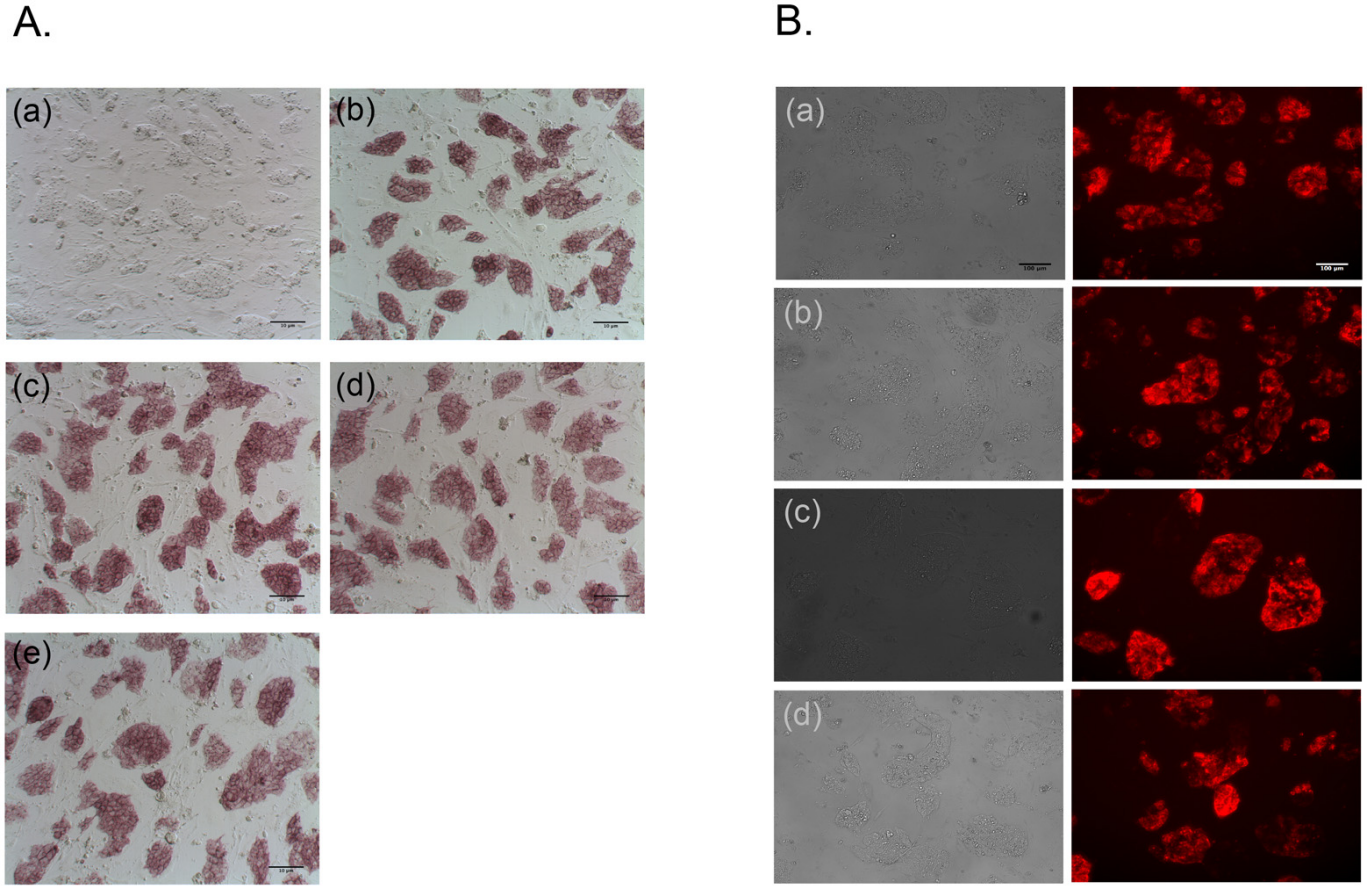
Characterization of stem cell properties of genetically modified, retrovirally transduced mESCs. (A) Alkaline phosphatase (AP) staining. Red-colored areas indicate alkaline phosphatase activity. (a). Bright field image of mESCs; AP staining: (b). mESCs; (c). mESC-H*ras*V12; (d). mESC-SV40 LTg; (e). mESC-H*ras*V12/SV40 LTg. Scale bare is 10 μm. (B) Immunocytochemistry staining of live-cells expressing SSEA1. Pairs of bright filed and SSEA1 images: (a). mESCs; (b). mESC-H*ras*V12; (c).mESC-SV40 LTg; (d). mESC-H*ras*V12/SV40 LTg. The representative images were obtained from fluorescent microscope with TRITC filter. Scale bar is 100 μm.

To confirm the stemness of transformed mESCs, the expression of alkaline phosphatase (AP) and stage-specific embryonic anitigen-1 (SSEA-1) stem cell marker was evaluated. The transformed mESCs (Fig 4A-(c)—(e)) expressed similar levels of AP staining compared to naïve (non-transformed) mESCs (Fig 4A-(b)) indicating uninterrupted maintenance of undifferentiated cells retaining their self-renewal potential. During further verification of the undifferentiated state of transformed mESCs with SSEA-1 specific antibody, transformed mESCs (Fig 4B-(b)—(d)) showed a positive for this surface marker as like non-transformed mESCs (Fig 4B-(a)). The detection of both AP and SSEA-1 in transformed mESCs demonstrated that transformation procedures did not seem to affect the stemness of mESCs.

### Characterizations of bioengineered cancer-like stem cells

**Fig 5:**
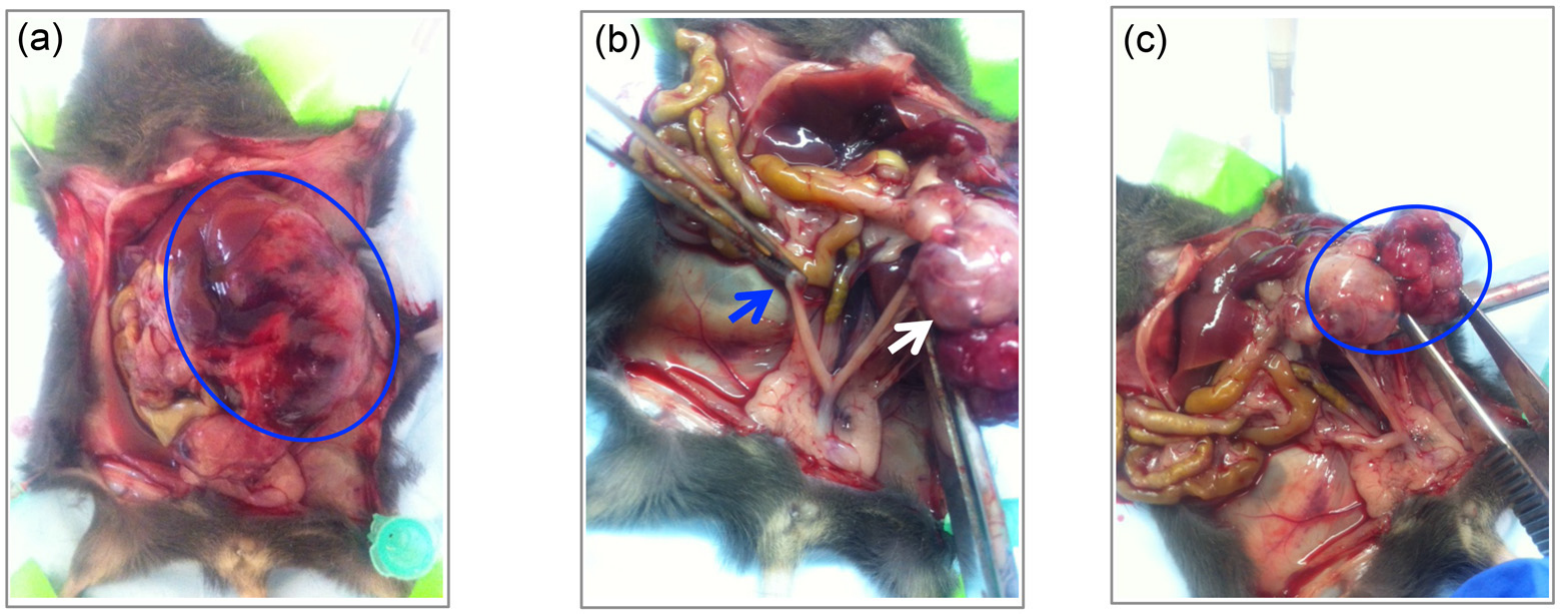
Ovarian mass at 31 days after orthotopic inoculation of mESCs transformed by both retroviral HrasV12 and SV40 LTg (mESC-HrasV12/SV40 LTg) into ovarian bursa of C57BL/6 mice. (A). Abdominal site with ovarian mass (blue circle); (B). Cervix with Uterus, right normal ovary (blue arrow) and left ovarian mass (white arrow); (C). Left ovary with partially intact capsule and mass breaking through the ovarian surface (blue circle).

To confirm the oncogenic potential of reprogrammed mESCs, mESC-H*ras*V12/SV40 LTg cells were either orthotopically inoculated to the left ovarian bursa or cleared inguinal mammary fat pads. At 31 days post inoculation, an ovarian mass had formed in mice that underwent orthotopic inoculation of the ovarian bursa. In contrast, mice having undergone orthotopic inoculation of the inguinal mammary fat pad did not form any masses. The abdominal situs in mice following ovarian inoculation demonstrated an ovarian mass (Fig 5A) in form of enlarged left ovary compared to the normal contra-lateral, non-injected right ovary (Fig 5B). Upon additional gross examination, the injected left ovary also visibly demonstrated the formed mass breaking through the ovarian surface as well as attaching itself to the peritoneal sidewall. The retroperitoneal space was also found to be occupied by the mass adhering to the back wall, and left kidney (Fig 5C). There was no gross evidence for abdominal metastases arising from this ovarian mass.

**Fig 6:**
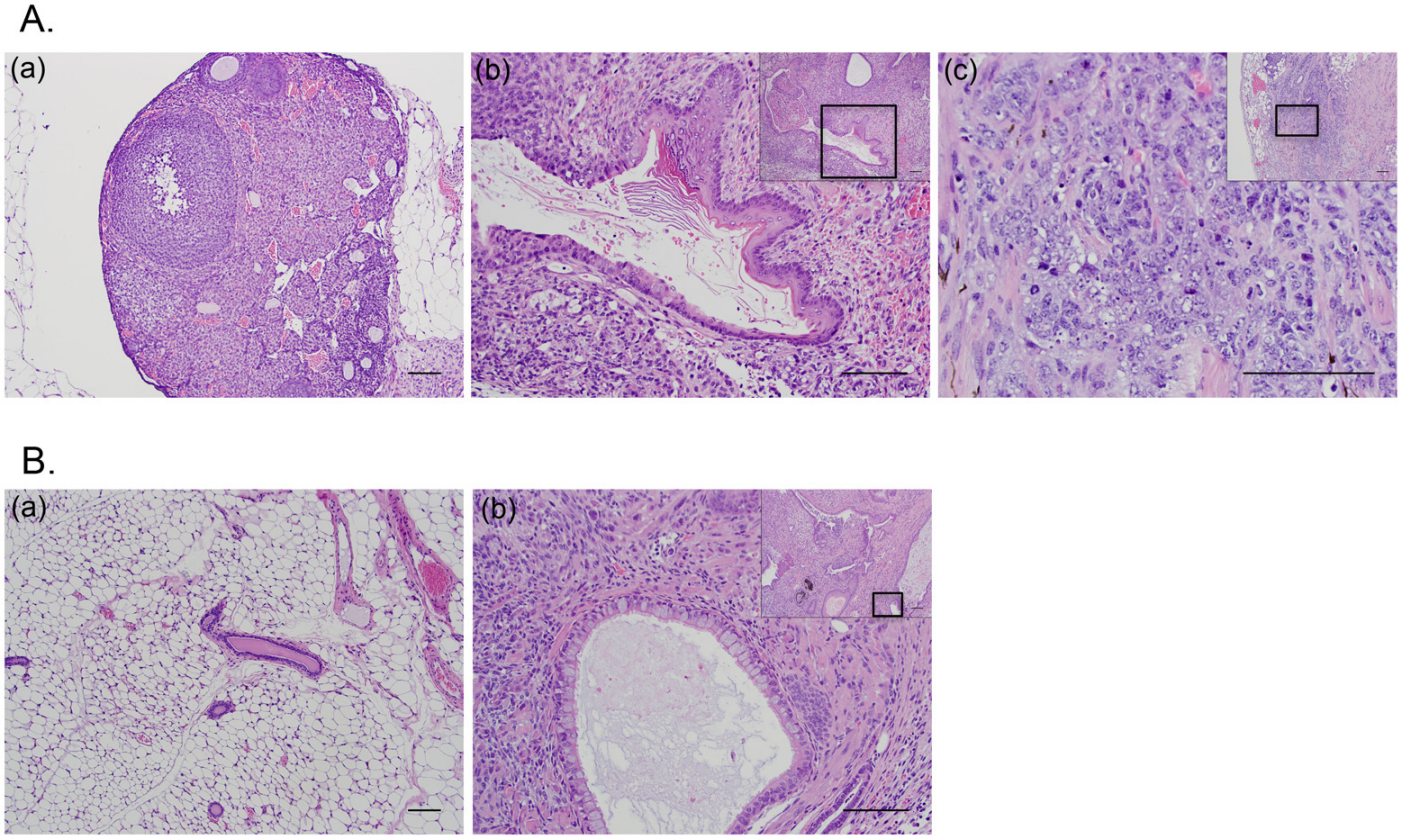
Histo-Pathological analysis of representative images (hematoxylin-eosin (H&E) tissue stain). (A) Ovarian Panel: (a). Normal right (non-injected) ovary (100X). Scale bar is 100 μm; (b). Left Ovarian mass (following orthotopic inoculation with mESC) showing signs of a mature teratoma (small window, 100X) characterized with a focus of mature, keratinizing squamous epithelium within the area depicted inside the box (200X). Scale bar is 100 μm; (c). Ovarian mass (following orthotopic inoculation with mESC-H*ras*V12/SV40-LTg) showing signs of immature teratoma (small window, 100X) characterized with scattered foci of a high-grade malignant neoplasm within the area depicted inside the box (400X). Scale bar is 100 μm. (B) Breast Panel: (a). Normal murine mammary tissue (100X). Scale bar is 100 μm; (b). Breast mass (following orthotopic inoculation of mESC into cleared inguinal mammary fat pad) exhibiting signs of mature teratoma (small window, 100X) characterized with the respiratory-like epithelium having well formed cilia within the area depicted inside the box (200X). Scale bar is 100 μm.

The histo-pathological analysis of ovaries injected with mESC revealed the generation of a mature teratoma exhibiting well-differentiated squamous epithelium with keratinization and respiratory-like epithelium (Fig 6A-(b)) compared to histological features of normal ovary (Fig 6A-(a)). Interestingly, ovaries injected with mESC-H*ras*V12/SV40 LTg also formed mature teratomas, but in addition revealed parts of an immature teratoma containing scattered foci of a high-grade malignant neoplasm, characterized by malignant cells arranged linearly, in small clusters, and occasionally as rudimentary gland-like structures (Fig 6A-(c) small window). Higher magnification revealed these malignant cells to be poorly differentiated with high nucleus:cytoplasm ratios, markedly atypical nuclei with coarsely clumped chromatin, variably prominent nucleoli, and numerous mitotic figures (Fig 6A-(c)). Mice who had their inguinal mammary fat pads injected with mESCs appeared to have generated mature teratomas exhibiting well-differentiated squamous epithelium, also containing pancreatic tissue, and respiratory-like epithelium with well-formed cilia (Fig 6B-(b)) compared to the histological assessment of normal breast tissue (Fig 6B-(a). In contrast, the breast injected mESC-H*ras*V12/SV40 LTg did not show any notable histological differences when compared to the normal, non-injected breast tissue.

## Discussion

The present study demonstrates that mouse embryonic stem cells (mESCs) can be reprogramed into induced cancer like stem cells (iCLSC) by introduction of well defined oncogenic elements, (the simian virus 40 large T oncogene (SV40 LTg) and an oncogenic ras (H*ras*V12)) by using a mouse stem virus long terminal repeat (MSCV-LTR)-based retroviral plasmid. The *in vitro* reprogrammed mESCs exhibited enhancement of proliferation and maintenance of stem cell properties under *in vitro* culture conditions. In addition, those transformed cells also demonstrated site-specific differences of the orthotopic tumor formation of following inoculation of ovary and breast tissues in immune competent mice. Thus, we suggest that *in vitro* reprogramming of mESCs with oncogenic elements may be a potential approach to generate induced cancer like stem cells.

The MSCV-LTR retroviral system was found to be a potentially useful tool for the effective, oncogenic transformation of mESCs. Retroviral infection of mESCs seems highly dependent on viruses generated from different types of retroviral plasmids. In our MSCV-LTR retroviral system, we observed greater infection ability and maintenance of stable gene expression, when compared to the common Moloney virus-based retroviral system (i.e. pBABE series). Our recent findings are consistent with previous reports that retrovirus, which was generated from a MSCV-LTR-based retroviral plasmid, could maintain long-term and stable expression of genes in both embryonic stem (ES) cells and hematopoietic stem (HS) cells [21, 22].

*In vitro* reprogramming of mESCs to iCLSC met the requirements of well-defined oncogenic genes, H*ras*V12 and SV40 LTg, for both neoplastic transformation and anti apoptosis. Oncogenic ras mutation, which occurs in approximately 30% of all human tumors, is known to be involved in the process of neoplastic transformation of cells *in vitro* and has been well reported [13, 23, 24]. However, introduction of a constitutively active form of ras alone (i.e. H*ras*V12) showed its potential of sensitizing cells to apoptosis [24–26] and even induction of premature cell senescence through association of p53 accumulation [27]. In our proliferation assay with reprogrammed mESC, the lack of a significant enhancement of proliferation of the reprogrammed mESC with H*ras*V12 alone could be in part explained by the induction of either cellular senescence or apoptosis through the expression of a constitutively active form of H*ras*V12 alone. In contrast, mESC-SV40 LTg and mESC-H*ras*V12/SV40 LTg demonstrated their enhancing proliferative effects. Considering an anti-apoptotic role of SV40 LTg by inactivation of p53 [28], we suggest that the introduction of SV40 LTg into mESC may play a role in preventing mESC from premature cellular senescence or induction of apoptosis. Moreover, SV40-LTg could cooperate with H*ras*V12 during the neoplastic transformation of mESCs with prevention of H*ras*V12-induced cell death, which was found to be consistent with various previous reports [13, 29, 30].

The successful maintenance of stem cell properties in reprogrammed mESCs could be confirmed *in vitro* and *in vivo*. In the process of *in vitro* reprogramming of mESCs, we used retroviral infection to introduce oncogenic components. Although retroviral introduction of genes has the advantages of high infection and stable integration of exogenous genes into the host chromosomes, random insertion of retroviral components into host genomes cannot exclude the perturbation or loss of the expression of stem cell properties in mESCs. However, we did observe a stable expression of the two stem cell markers, alkaline phosphatase (AP) and SSEA-1, following retroviral transformation of mESCs. To gain a more comprehensive understanding of the mechanisms of the underlying this maintenance of stem gene expression following retroviral infection, further study is required. In addition to the maintenance of stem cell properties *in vitro*, orthotopic inoculation of reprogrammed mESCs *in vivo* also showed the formation of immature teratomas in the ovary of mice. In fact, the observed teratoma formation *in vivo*, including immature, malignant components also exemplified the successful maintenance of stem cell properties *in vivo* throughout the entire reprogramming process.

If the development of various malignant tumors from the stem cells will indeed be possible, this may require additional studies to further investigate the specific role of different microenvironments in tumorigenesis [31]. Our animal experiments allowed us to observe the differences of the teratoma formation in two different orthotopic implantation sites (ovary and breast). In orthotopic ovarian implantation, mESC-H*ras*V12/SV40 LTg induces the generation of immature teratoma exhibiting scattered foci of a high-grade malignant neoplasm. However, breast placed with the mESC-H*ras*V12/SV40 LTg did not show the formation of teratoma. Although the contribution of microenvironment in those two site for the generation of malignancy remains to be studied, a recent report by Yan T *et al* suggested the potential contribution of tumor-favorable microenvironmental factors during the transformation of mouse induced pluripotent stem cells (miPSCs) into cancer stem cells (CSCs) [32]. Therefore, we believe that ovarian microenvironment may be the place favorable for the advancement of the tumorigenesis of mESC- H*ras*V12/SV40 LTg compared to the place of breast.

In conclusion, we demonstrated the successful generation of induced cancer like stem cells by using oncogenic *in vitro* reprograming of mESCs. The reprogrammed mESCs were characterized through their oncogenic gene expression and unhampered maintenance of stem properties *in vitro* and *in vivo*. In addition, these modified cells showed the formation of teratomas containing immature, malignant parts when growing orthotopically at implantation site favorable for tumorigenesis. Limitations of this research exist based on the formation of advanced teratomas *in vivo* in regards to the issue of tumor- favorable microenvironments. Furthermore, we suggest the necessity of an aberrant microenvironment for the development and maintenance of tumors derived from induced cancer like stem cells. To better understand the processes of natural tumorigenesis, as well as to establish more predictable cancer animal models, continued study of the detailed roles the tumor microenvironment as well as the identification of potentially aberrant microenvironmental factors will be required to gain insights into the *in vivo* generation of various cancers derived from induced cancer like stem cells. Our work has provided pioneering groundwork to enable a variety of additional future research applications for these induced cancer like stem cells, but more research is needed to better understand underlying mechanisms of iCLSCs tumorigenesis.

## Supporting Information

S1 Table Tumor formation in mice. (TIF)
