# Supplementary figures and images for: Design and Characterization of Bioengineered Cancer-Like Stem Cells
Source: PLoS One. 2015 Oct 21;10(10):e0141172. doi: 10.1371/journal.pone.0141172 (PMC4619203; doi:10.1371/journal.pone.0141172)

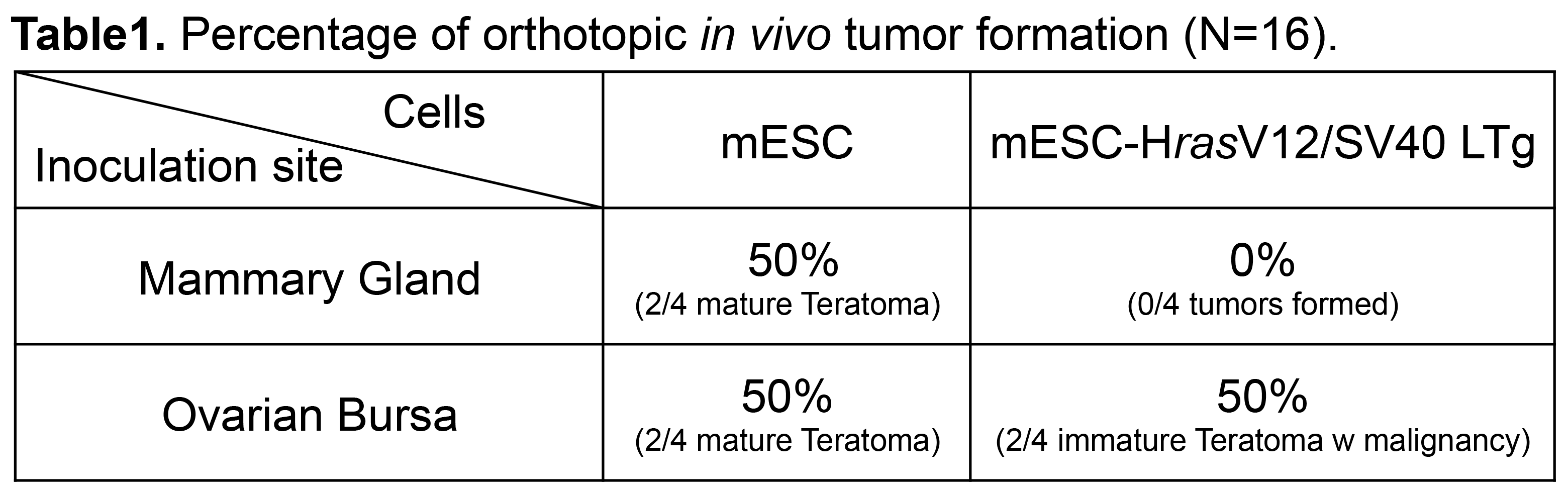

Supplement: S1 Table — (TIF) [file pone.0141172.s001.tif]
